# Lower pole renal stone: flexible ureteroscopy or shockwave lithotripsy? The anatomy is the key

**DOI:** 10.1590/S1677-5538.IBJU.2018.02.01

**Published:** 2018

**Affiliations:** Professor Titular, Unidade Urogenital da Univ. Est. do Rio de Janeiro - UERJ, RJ, Brasil; Urologista do Hospital da Lagoa Federal, Rio de Janeiro, RJ, Brasil; Editor Associado do International Braz J Urol

The March-April 2018 issue of the International Braz J Urol presents original contributions with many interesting papers in different fields: BPH, Renal stones, Prostate Cancer, Renal Cell Carcinoma, Bladder Cancer, Uretrhal Strictures, Prostatitis, Urinary Incontinence, Urinary Tract Infections, Ureteropelvic Junction Obstruction, Laparoscopy and Renal Anomalies. The papers come from many different countries such as Brazil, USA, Turkey, China, Italy, Lebanon, Argentina, Spain, Canada, Thailand and India, and as usual the editor's comment highlights some papers. We decided to comment the paper about a very interesting topic: The treatment of the lower pole stones.

Doctor Ozgor and collegues from Turkey performed on page **314** an interesting study about the management of 10-20 millimeter lower pole renal stone. The authors studied 241 patients with 10-20 millimeter lower pole stone (LPS) treated with lithotripsy (SWL) - 113 patients and flexible ureterorenoscopy (f-URS) - 128 patients. The authors analyzed the medium term follow-up results of these procedures. The stone-free rate was 77.9% (88/113 patients) for the SWL group and 89% (114/128 patients) for the f-URS group (p=0.029). Stone recurrence was detected in 28 (35.4%) patients in SWL group and in 17 (17.2%) patients in f-URS group (p=0.009). Stone types and 24 hour urine sample results were similar between groups (p=0.123 vs p=0.197, respectively). Multivariate regression analysis revealed that f-URS procedure and absence of abnormality in 24 hour urine analysis significantly decreased stone recurrence in medium term follow-up (p=0.001 and p<0.001, respectively). The authors concluded that patients which underwent f-URS for LPS, faced less stone recurrence, independent from diet regimen and metabolic evaluation in medium term follow-up.

This paper is very interesting but the authors do not analyze the anatomic factors of the lower pole in the two groups studied. The lower pole stones can be treated through the ESWL, FUR, and percutaneous nephrolithotripsy (1). The anatomical aspects of the lower pole, especially considering distribution of calices, the angle between the infundibulum and the renal pelvis, the length of the infundibulum and the diameter of the calices are crucial to determining the success of each type of chosen treatment (2-6). The classification of the renal collector system is in accordance to the distribution of calices in the mid-kidney area as it is well accepted in the reference literature (7, 8) and recent papers shows that the collector system with kidney midzone drained by crossed calices presented the lower rate of accessibility during FUR (9-11). Knowledge of the anatomy of the renal collector system has great importance for the strategy of the surgeon during the FUR and ESWL. Several studies in the referenced literature correlate anatomical parameters such as LIP, the length and width of the lower infundibulum as prognostic factors for the success of the procedure (2-8). In a recent study, it was shown that patients with a high infundibular length or a very acute LIP have a greater chance of requiring a second procedure but without influence on the rate of complications (5).

Patients with unfavorable anatomical factors have lower rates of success in FUR and ESWL, so we always need to study the anatomical aspects of the lower pole before the treatment of lower pole stones.
